# Stacked ensemble model for NBA game outcome prediction analysis

**DOI:** 10.1038/s41598-025-13657-1

**Published:** 2025-08-16

**Authors:** Guangsen He, Hyun Soo Choi

**Affiliations:** Present Address: Department of Physical Education, Hanyang University, 222, Wangsimni-ro, Seongdong-gu, Seoul, Republic of Korea

**Keywords:** Machine learning, Stacked ensemble, Shapley additive explanation (SHAP), Features selection, Data mining, Mathematics and computing, Applied mathematics, Computer science

## Abstract

This research presents a stacked ensemble approach that employs artificial intelligence (AI) techniques to predict the outcomes of NBA games. Several machine learning algorithms were utilized, including Naïve Bayes, AdaBoost, Multilayer Perceptron (MLP), K-Nearest Neighbors (KNN), XGBoost, Decision Tree, and Logistic Regression. The best-performing models were selected to serve as the base learners in the ensemble architecture. To improve the model’s interpretability and transparency, SHAP was used to clarify its decision-making process. The model was trained and evaluated using publicly available NBA datasets from 2021–2022,2022–2023, and 2023–2024. Experimental results indicate that the proposed ensemble approach is practical in predicting game outcomes. Furthermore, the SHAP analysis provides valuable insights into the underlying predictive mechanisms, offering actionable information for coaches and analysts.

**Supplementary Information:**

The online version contains supplementary material available at 10.1038/s41598-025-13657-1.

## Introduction

The National Basketball Association (NBA), comprising 30 teams, is among the most popular and commercially influential professional sports leagues worldwide. Given its global reach and competitiveness, NBA game outcomes draw widespread attention from fans, the media, the betting industry, and sports professionals^1^. Over its nearly 70-year history, the NBA has accumulated a vast repository of structured data, including statistics on points, rebounds, assists, and other key performance indicators.

The increasing availability of data, along with advancements in analytical tools, has sparked a growing interest in data-driven analysis within basketball^2^. The rapid development of artificial intelligence (AI) and computational power has enabled the integration of machine learning (ML) techniques into sports analytics, making it a prominent trend in research. ML is highly effective for modeling complex nonlinear interactions and uncovering hidden patterns in large-scale, high-dimensional datasets. Compared to traditional statistical models, ML approaches offer enhanced predictive capabilities and adaptability. By selecting suitable algorithms that align with specific objectives and data characteristics, researchers can significantly enhance both predictive performance and model interpretability, thereby overcoming the limitations of conventional methods.

Numerous predictive and statistical models, including various ML techniques, have been developed to forecast NBA game outcomes^3^. With the rapid advancement of ML algorithms, modern systems can uncover complex data patterns, classify instances, make accurate predictions, and support informed decision-making processes. The ultimate goal is to train models that outperform human intuition in specific predictive tasks. These models are commonly optimized through supervised learning techniques and tested on separate datasets to evaluate their generalization ability to unseen data^4–6^. Various modeling techniques have been proposed in sports analytics to enhance predictive performance for game outcomes^7–9^.

In this study, we utilize regular-season NBA data from 2021 to 2022 to 2023–2024 and apply a diverse set of machines learning algorithms, including Naive Bayes, AdaBoost, Multilayer Perceptron (MLP), K-Nearest Neighbors (KNN), XGBoost, Decision Tree, and Logistic Regression. These models serve as candidate-based learners for an ensemble framework. We adopt a 5-fold cross-validation procedure to ensure robustness and generalizability and perform hyperparameter tuning via grid search. We rely on five key metrics to evaluate model performance: Accuracy, Precision, Recall, F1-score, and AUC. Based on the experimental results, the top-performing base classifiers are selected and integrated into a Stacked ensemble model, with an MLP neural network as the meta-learner. This approach is designed to leverage the strengths of heterogeneous learners and maximize predictive accuracy.

Furthermore, we enhance model interpretability using SHAP (Shapley Additive Explanations), which provides both global and instance-level insights into feature importance. This not only enhances the transparency of the model but also provides actionable insights for coaches, analysts, and stakeholders seeking data-driven support in strategic decision-making. These interpretability results offer not only theoretical insights but also practical value—providing strategic support to coaches for real-time decisions and aiding sports bettors, analysts, team managers, and sponsors in understanding outcome determinants from a data-driven perspective. The contributions of this study are primarily reflected in the following aspects:

- Unlike the majority of existing studies that rely on a single predictive model, this study develops a stacked ensemble model that integrates multiple base learners (such as LGBM, XGBoost, and Random Forest) with a meta-learner. This architecture leverages the complementary strengths of different algorithms, thereby enhancing both prediction accuracy and generalization capability.
- By incorporating the SHAP method, the study enables interpretability analysis of model outputs. This ensures that the predictions are not only accurate but also interpretable, significantly increasing the model’s practical utility in sports theory and tactical decision-making.
- While most sports data studies primarily apply traditional machine learning or deep learning techniques, few have systematically adopted an interpretable ensemble framework combining stacking and SHAP analysis. This research bridges that methodological gap by proposing a novel, explainable architecture for NBA game prediction, contributing both theoretical innovation and practical relevance to the field.

This paper is structured as follows: the Related Work section reviews previous research on NBA game prediction and the implementation of machine learning within sports analytics. The Materials and Methods section presents the research methodology, covering data preprocessing, feature engineering, model selection, and evaluation techniques. In the Results section, the experiment findings are detailed, along with an assessment of the model’s predictive performance. The Discussion section offers a critical analysis of the model’s advantages, limitations, and areas for possible improvement. Finally, the Conclusions and Future Work section summarizes key insights and proposes directions for subsequent studies.

## Related work

While machine learning has been applied in various areas of sports analytics, there remains a need to enhance the effectiveness of predictive models developed using this innovative technology. In this section, we will examine the techniques proposed by researchers to improve the accuracy of game outcome predictions, along with an analysis of their advantages and disadvantages. This section establishes the foundation for the Stacked Ensemble Machine Learning Approach proposed in this study by reviewing existing advancements and identifying research gaps in the field. A thorough examination of related research enables readers to gain a deeper understanding of the current state of game outcome prediction. It highlights the significance of our approach in advancing this field of study.

Ahmadalinezhad and Makrehchi^10^. introduced a novel metric known as the Inverse Square Metric (ISM) and leveraged an edge-centric multi-view network to predict lineup performance in specific game contexts. Their experimental results showed that the ISM method achieved an average accuracy of 68%, while the edge-centric multi-view approach reached a notably higher accuracy of 80%.

Balli and Özdemir^11^. traditional machine learning techniques were applied to classify basketball match outcomes, utilizing algorithms such as K-Nearest Neighbors (KNN), Logistic Regression, Multilayer Perceptron (MLP), Naïve Bayes, J48 Decision Tree, and Voting. Among these methods, Multilayer Perceptron (MLP) achieved the highest prediction accuracy of 98.90%, highlighting its effectiveness in modeling complex game dynamics.

Caliwag et al.^12^ proposed an enhanced basketball game prediction model based on a Cascading Algorithm, which integrates Naïve Bayes, Four Factor Analysis, and Fuzzy Logic Algorithms. Their model demonstrated an accuracy range of 69–70%, showcasing the potential of hybrid analytical frameworks.

Several studies have also compared the performance of various traditional machine learning algorithms in real-time basketball game prediction. Numerous studies have applied hyperparameter optimization techniques to enhance the performance of classification models, including KNN, LightGBM, SVM, Random Forest, Logistic Regression, Decision Tree, and XGBoost. Ouyang et al.^13^ found that XGBoost consistently outperformed other models across multiple benchmark tests.

Zhao et al.^14^ proposed a method that uses Graph Neural Networks (GNNs) to predict the outcomes of basketball games by transforming structured match data into graph representations suitable for unstructured learning. Their method effectively captured complex player passing interactions, integrating Graph Convolutional Networks (GCN) with machine learning algorithms. The proposed graph-based approach attained a prediction accuracy of 71.54%, highlighting its effectiveness and potential applicability within the domain of sports analytics.

Osken and Onay^15^ employed K-means and C-means clustering techniques to categorize NBA players based on their playing styles. Using these clusters and game-specific features, they built an Artificial Neural Network (ANN) model to forecast game outcomes. The model, evaluated on NBA data from the 2012 to 2018 seasons, achieved an accuracy of 76%.

Çene^16^ examined the predictive performance of seven machine learning algorithms on European league basketball games spanning the 2016–2017 to 2020–2021 seasons. The analysis identified Logistic Regression, SVM, and ANN as the leading models, achieving an overall prediction accuracy of approximately 84%.

Harman Deep and Sushma^17^ explored NBA game predictions for the 2015–2016 season, applying Support Vector Machines (SVM) and Fuzzy Hybrid SVM. Their results demonstrated that SVM was the most effective model, achieving an accuracy of 88.26%.

Similarly, Ayyıldız^18^ utilized Artificial Neural Networks (ANN) to forecast NBA game outcomes for the 2015–2016 season, achieving an impressive accuracy of 90%. This further demonstrates the effectiveness of deep learning methods in sports analytics.

Horvat et al.^19^ evaluated the effectiveness of seven different machine-learning classification algorithms for predicting the outcomes of basketball matches. Their findings indicated that KNN performed the best, with an accuracy of 60.01%, highlighting the model’s potential despite its relatively lower performance than other approaches. Table 1 provides a summary of some studies.

**Table 1:** Summary of some studies related to water quality classification.

Study	Dataset	Method(s)	Highest Accuracy
Ahmadalinezhad & Makrehchi (2020)^10^	NBA 2007–2019	ISM, Edge-centric multi-view	68% (ISM), 80% (Multi-view)
Balli & Özdemir (2021)^11^	European league2012-2017	KNN, LR, MLP, NB, J48, Voting	98.90% (MLP)
Caliwag et al. (2018)^12^	NBA 2015–2016	Cascading NB, Four Factor, Fuzzy Logic	69–70%
Zhao et al. (2023)^14^	NBA 2012–2018	GCN + ML	71.54%
Osken & Onay (2022)^13^	NBA 2012–2018	K-means/C-means + ANN	76%
Çene (2022)^16^	European league 2016–2021	LR, SVM, ANN	84%
Harman Deep & Sushma (2017)^17^	NBA 2015–2016	SVM, Fuzzy Hybrid SVM	88.26%
Horvat et al. (2020)^19^	NBA 2009–2018	Logistic R, Naive Bayes, Decision Tree, Multilayer Perceptron, Random Forest, KNN, LightBoost	60.01%(KNN)

Although various machine learning techniques—such as KNN, SVM, Decision Trees, XGBoost, ANN, and Naïve Bayes—have been widely applied in existing research on basketball game outcome prediction, most of these studies focus on the construction or performance comparison of individual models. A systematic integration of multiple learners to enhance generalization and robustness remains a significant gap.

In contrast, this study employs a stacking ensemble learning strategy, wherein multiple classical models are used as base learners in the first layer, and a MLP is utilized as the meta-learner to make the final prediction. Compared to traditional single-model approaches, the stacking method effectively leverages the complementary strengths of diverse algorithms, reduces the risk of overfitting inherent in single models, and improves the model’s adaptability to varying feature combinations.

While some recent studies have combined deep learning or neural network-based methods—for example, Zhao et al.^12^ achieved 71.54% accuracy using Graph Convolutional Networks (GCNs)—such methods often entail high computational complexity, making them difficult to implement in small-sample or resource-constrained environments. By contrast, the method proposed in this study features a clear model structure, moderate training cost, and improved practicality and interpretability.

Furthermore, due to differences in datasets and feature variables, the optimal predictive model can vary significantly across studies. For instance, high-accuracy models proposed by Balli & Özdemir^9^ and Çene^14^ were developed based on European basketball leagues. Given the structural and tactical differences between the NBA and European leagues, the applicability and effectiveness of these models on NBA datasets remain uncertain. To address this gap, the present study systematically compares high-performing models from the literature. It introduces a more advanced stacking ensemble learning algorithm that integrates multiple classifiers to enhance prediction accuracy. This approach has not been widely applied in basketball analytics. It thus offers a novel methodological perspective for predicting NBA game outcomes, contributing both innovation and theoretical advancements to the field.

## Materials and methods

**Fig. 1:**
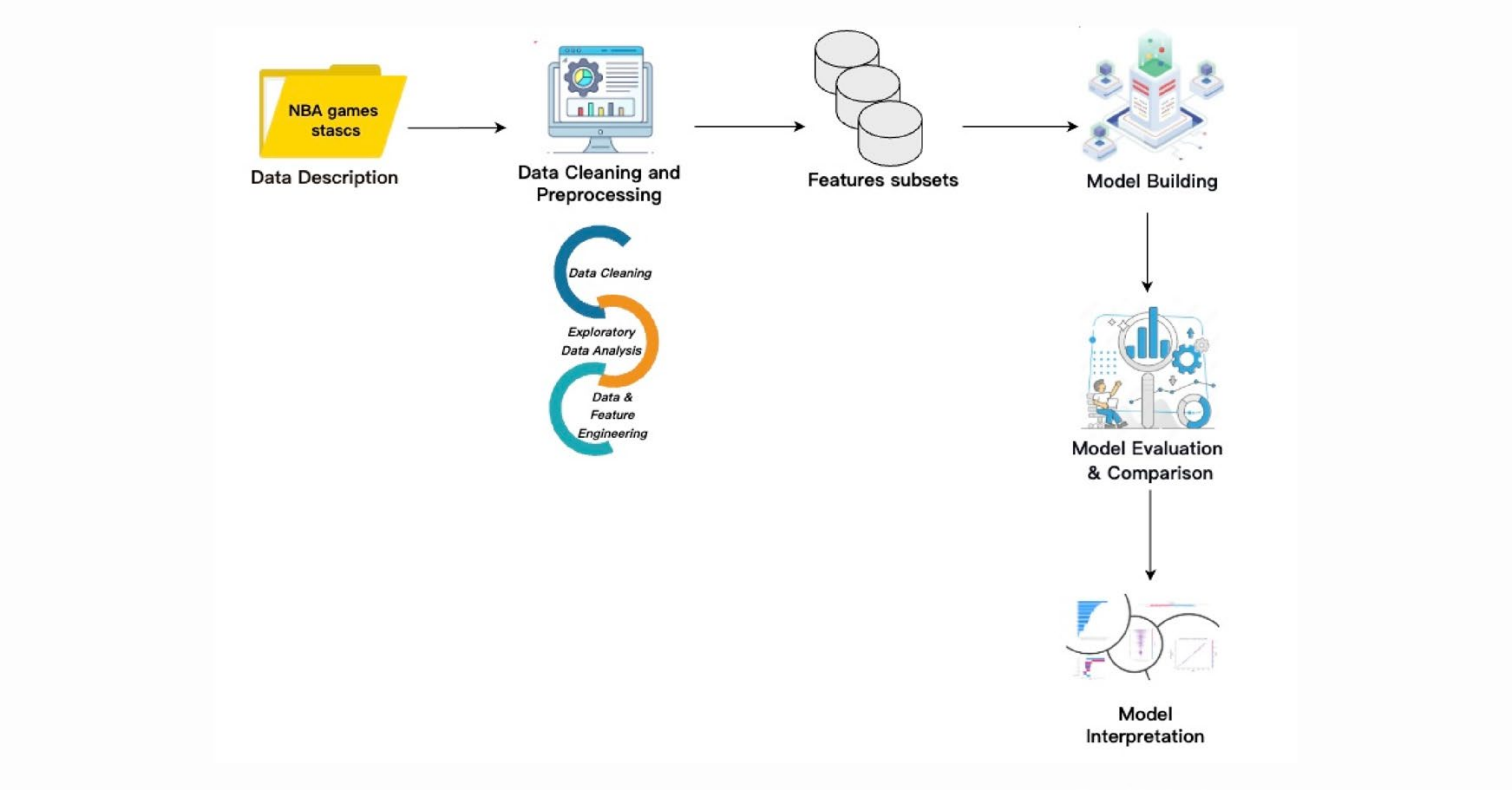
The proposed framework for classifying NBA game outcomes.

This study aims to develop a predictive framework for NBA game outcomes using a machine-learning strategy based on the Stacking ensemble method. This section details the materials and methods used to achieve our objective. Figure 1 displays the overall workflow of the proposed framework. The entire experimental procedure was carried out in a Jupyter Notebook environment using Python. We utilized several key Python libraries—including NumPy, Pandas, Scikit-learn, and Matplotlib—for data processing, model development, and result visualization.

### Dataset

The data used in this study were obtained from the official NBA website (https://www.nba.com), which provides a comprehensive collection of multi-dimensional information, including basic player and team statistics, advanced efficiency metrics, spatial tracking data, lineup performance, and salary and management records. These datasets are widely used in both tactical analysis and machine learning modeling.

This study focuses primarily on game-level performance and outcomes from the regular seasons of the 2021–2022, 2022–2023, and 2023–2024 NBA seasons. Specifically, data were collected from all 1,230 games per season, totaling 3,690 games and 7,380 samples (accounting for both home and away teams). The dataset comprises 20 feature variables, including total field goals made, three-point shots made, and other offensive and defensive indicators. The game outcome (win or loss) serves as the target variable. Detailed descriptions of all variables are provided in Table 2.

**Table 2:** Summary of feature Variables.

Variable name	Description
Team	The name of the team
Opponent team	A player against another player
MP	Total number of minutes played
FG	Field goals made
FGA	Field goal attempts
FGP	Field goal percentage (A statistic calculation by dividing field goals made by field goal attempts)
3P	A field goal in a basketball game made from beyond the three-point line
3PA	Three-point attempts (A field goal in a basketball game made from beyond the three-point line)
3PP	Three-point percentage (Ratio of field goals made to field goals attempted)
2P	A field goal in a basketball game made from inside the three-point line.
2PA	A field goal in a basketball game attempted from inside the three-point line.
2PP	Two-point percentage (Ratio of two-point field goals made to two-point field goals attempted)
FT	Free throws made
FTA	Free throws attempted
FTP	Free throw percentage
ORB	Offensive rebound (Statistic that measures a player’s number of offensive rebounds in proportion to the total number of offensive rebounds available during active play.)
DRB	Defensive rebounds (Statistic that measures a player’s number of defensive rebounds in proportion to the total number of defensive rebounds available during active play.)
TRB	Total rebounds (Rebounds are usually grabbed by the front-court players, but guards are also expected to rebound the ball when it falls far from the rim.)
AST	Assist (An assist is attributed to a player who passes the ball to a teammate in a way that leads to a score by field goal.)
STL	Steal (A steal occurs when a defensive player legally causes a turnover by his positive, aggressive action)
BLK	Blocks (A block occurs when a defensive player legally deflects a field goal attempt from an offensive player to prevent a score)
TOV	Turnovers
PF	Personal fouls
Results	1 = win; 0 = loose

#### Data processing

To facilitate model training, the game outcome variable for all regular-season games across the three NBA seasons was encoded as a binary classification variable, where a win was labeled as 1 and a loss as 0. This binary encoding allows machine learning models to distinguish between game outcomes more effectively.

As shown in the summary statistics presented in Table 3, the resulting dataset is balanced, containing an equal number of win and loss samples. Specifically, among the 7,380 total samples, there are 3,690 observations for each class (win and loss).

**Table 3:** Summary statistics of class Distribution.

Variables	Number of Samples (Games)	Total Sample Size (Games)
WIN (1)	3690 (1845Games)	7380 (3690 Games)
LOSS (0)	3690 (1845Games)	

#### Dataset partitioning

To prevent model overfitting and ensure robust predictive performance, the dataset was randomly partitioned into training and testing subsets. Given that each regular-season game across the three NBA seasons is mutually independent, random sampling was employed for the split.

Specifically, 80% of the data was allocated to the training set, and the remaining 20% to the testing set. The training set was used for model construction, while the testing set was used for evaluating the model’s performance. The detailed distribution of samples is presented in Table 4.

**Table 4:** Sample distribution between training and testing Sets.

Total Sample Size (Games)	Number of Training Samples (Games)	Number of Testing Samples (Games)
7380 (3690 Games)	5904 (2952 Games)	1476 (738 Games)

### Feature selection

**Fig. 2:**
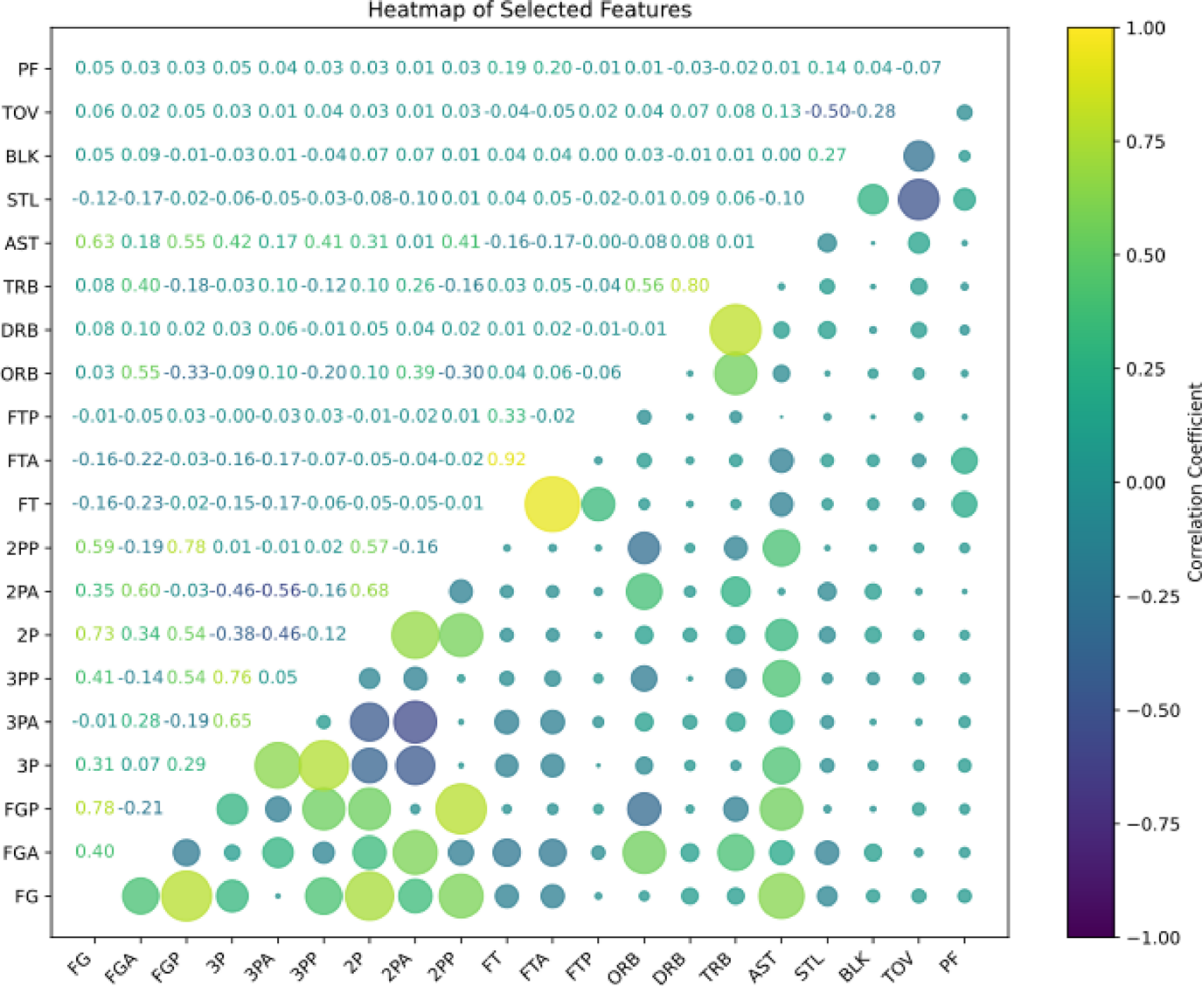
Pearson Correlation Heatmap of Feature Variables.

Feature selection is a crucial step that directly impacts the accuracy and generalization performance of a model^20^. To prevent the issue of multicollinearity among variables, which can reduce model interpretability, key features were further screened to address this concern. We employed Exploratory Data Analysis (EDA) techniques and calculated the Pearson correlation coefficients between feature variables to assess inter-variable relationships visually. As shown in the correlation heatmap in Fig. 2, no severe multicollinearity was observed among the 20 feature variables. Therefore, all 20 features were retained for model training and analysis. Descriptive statistics for each variable are presented in Table 5.

**Table 5:** Descriptive statistics of feature Variables.

Indicators	Mean	Std	Min	0.25	0.5	0.75	Max
result	0.5	0.5	0	0	0.5	1	1
FG	41.59	5.27	23	38	41	45	65
FGA	88.43	7.08	64	84	88	93	121
FGP(%)	47.13	5.53	27.7	43.3	47.1	50.7	68.7
3P	12.54	3.85	2	10	12	15	27
3PA	34.83	6.72	12	30	34	39	63
3PP(%)	35.91	8.37	6.9	30.3	35.7	41.4	64.5
2P	29.05	5.38	11	25	29	33	50
2PA	53.6	8.16	24	48	53	59	90
2PP(%)	54.38	7.39	28.07	49.12	54.24	59.26	81.08
FT	17.45	5.83	0	13	17	21	44
FTA	22.37	6.97	0	17	22	27	52
FTP(%)	78.01	10.1	0	71.4	78.6	85	100
ORB	10.44	3.88	0	8	10	13	29
DRB	33.37	5.4	16	30	33	37	60
TRB	43.81	6.64	23	39	44	48	74
AST	25.55	5.08	8	22	25	29	50
STL	11.78	4.66	0	8	12	15	30
BLK	6.69	2.98	0	5	6	9	21
TOV	7.66	5.14	0	4	6	11	29
PF	19.45	4.1	4	17	19	22	35

Std = standard deviation; Min = minimum; 0.25 = lower quartile; 0.5 = median; 0.75 = upper quartile; Max = maximum.

### Model building

After completing data preprocessing, this study utilizes the Scikit-Learn Python toolkit to train machine learning models. The dataset is initially divided into training and testing sets, ensuring a systematic approach to model evaluation. Various machine learning classifiers are then applied to train and evaluate the model’s performance, allowing for a thorough comparison of their predictive abilities.

To construct the baseline models, each candidate machine learning algorithm is evaluated using 5-fold cross-validation, a widely accepted technique to ensure robust and unbiased performance estimation. The model yielding the highest average accuracy across folds is selected for inclusion in the base layer of the Stacking ensemble framework. The accuracy metric computed via 5-fold cross-validation is formally defined in Eq. 1.

1 $$\:C\:{A}_{k}=\frac{\sum\:_{i=1}^{k}{A}_{i}}{k}$$

In this context, *k* represents the number of cross-validation folds, while *Ai* indicates the accuracy achieved on the *i*-th fold. The resulting *CA*_*k*_ represents the model’s average cross-validated accuracy. According to the evaluation results above, the models with the highest accuracy are selected to form the base layer of the Stacked Ensemble. The selection criterion is further specified in Eq. 2.

2 $$\:\forall \:\{ i \in \:N,0 < i < n\} ,\:model_{i} \: = \:O_{{(max \ldots \:min)}} (CA_{1} ,CA_{2} , \ldots \:,CA_{n} )$$

Let model*i* represent the selected model, while O (max…min) indicates the descending order function based on the cross-validation accuracies of n different models. The top-performing models are chosen to form the foundation of the proposed Stacked Ensemble Model.

#### Selected models

##### Multilayer perceptron

The Single-Layer Perceptron is limited in its ability to solve problems that are not linearly separable. The MLP was developed as an extension to address this limitation. The MLP incorporates a multilayer network architecture trained using the backpropagation algorithm. The architecture generally includes three-layer types: the input layer, one or more hidden layers, and the output layer^21^. In contrast to the Single-Layer Perceptron, the MLP includes at least one intermediate (hidden) layer that enables the model to capture non-linear relationships^22^.

##### XGBoost

XGBoost is an ensemble learning algorithm that generates predictions by aggregating the outputs of multiple base learners, typically decision trees^23^. While it shares structural similarities with Random Forest in using decision trees as fundamental components, XGBoost offers notable advantages over traditional Gradient Boosting Decision Tree (GBDT) methods. These include significantly faster computational speed, improved generalization performance, and superior scalability, making it well-suited for large-scale and high-dimensional machine learning tasks. Unlike many machine learning frameworks, XGBoost offers extensive support for fine-tuning regularization parameters, providing greater control over model complexity and improving overall performance^24^. Due to its efficiency and strong generalization capability, XGBoost has become a widely used technique for predictive modeling across various domains, including sports analytics and NBA game outcome prediction.

##### AdaBoost

AdaBoost is an ensemble learning method that builds a strong classifier by iteratively combining multiple weak learners. In each iteration, more focus is placed on instances that were misclassified in the previous round, guiding subsequent classifiers to concentrate on more challenging samples. AdaBoost classifiers operate as meta-estimators, initially training a base learner on the original dataset^25^. In the following iterations, the same base learner is retrained multiple times but with adjusted sample weights—specifically, increasing the weights of previously misclassified instances—so that the model progressively improves its performance on complex examples.

##### Naive Bayes

This classification algorithm, named after the English mathematician Thomas Bayes, is based on Bayes’ theorem, and relies on probabilistic reasoning. It performs classification by applying probability calculations derived from training data and assigns a new instance to the class with the highest posterior probability^24^. The mathematical formulation of Bayes’ theorem is presented in Eq. 3.

3 $$\:P\left(A|B\right)=\:\frac{P\left(B|A\right)\times\:P\left(A\right)}{P\left(B\right)}$$

P (A | B) represents the probability of event A occurring, given that event B has already occurred.

P (B | A) denotes the likelihood of event B occurring, given that event A has occurred.

P(A) and P(B) refer to the prior (unconditional) probabilities for events A and B, respectively.

##### K-Nearest neighbor (KNN)

The K-nearest neighbor (KNN) algorithm is a supervised learning technique used for both classification and regression tasks. It is a non-parametric, instance-based learning algorithm that makes predictions based on the similarity between data points. In the classification process, when a new sample needs to be categorized, the algorithm identifies the k closest data points from the training set based on a chosen distance metric (e.g., Euclidean distance, Manhattan distance, or Minkowski distance). The majority class among these K nearest neighbors is then assigned as the class label for the new sample^26^.

##### Logistic regression

Logistic Regression is a regression-based classification method primarily designed for binary classification problems. It can be considered an extension of Linear Regression, optimized to estimate the probability that a given observation belongs to a particular category. In other words, Logistic Regression predicts the likelihood of an outcome based on a set of independent variables and then classifies the dependent variable accordingly^26^.

##### Decision tree

A Decision Tree is a supervised learning model for classification and regression tasks. It follows a hierarchical, tree-like structure to recursively partition the dataset based on feature values, ultimately reaching a leaf node representing the predicted outcome^27,28^.

#### Proposed stacked ensemble model

This study proposes a Stacked Ensemble (Stacking) model to enhance overall performance by integrating predictions from multiple base models. Stacking enables the training of diverse machine learning models to solve similar problems, combining their outputs to construct a more robust and accurate predictive model^28,29^. In contrast to ensemble techniques like Bagging and Boosting, Stacking emphasizes the integration of heterogeneous and strong base models, utilizing their complementary capabilities to enhance overall predictive performance. By aggregating the predictions of multiple models, the approach enhances generalization ability and performance stability.

**Fig. 3:**
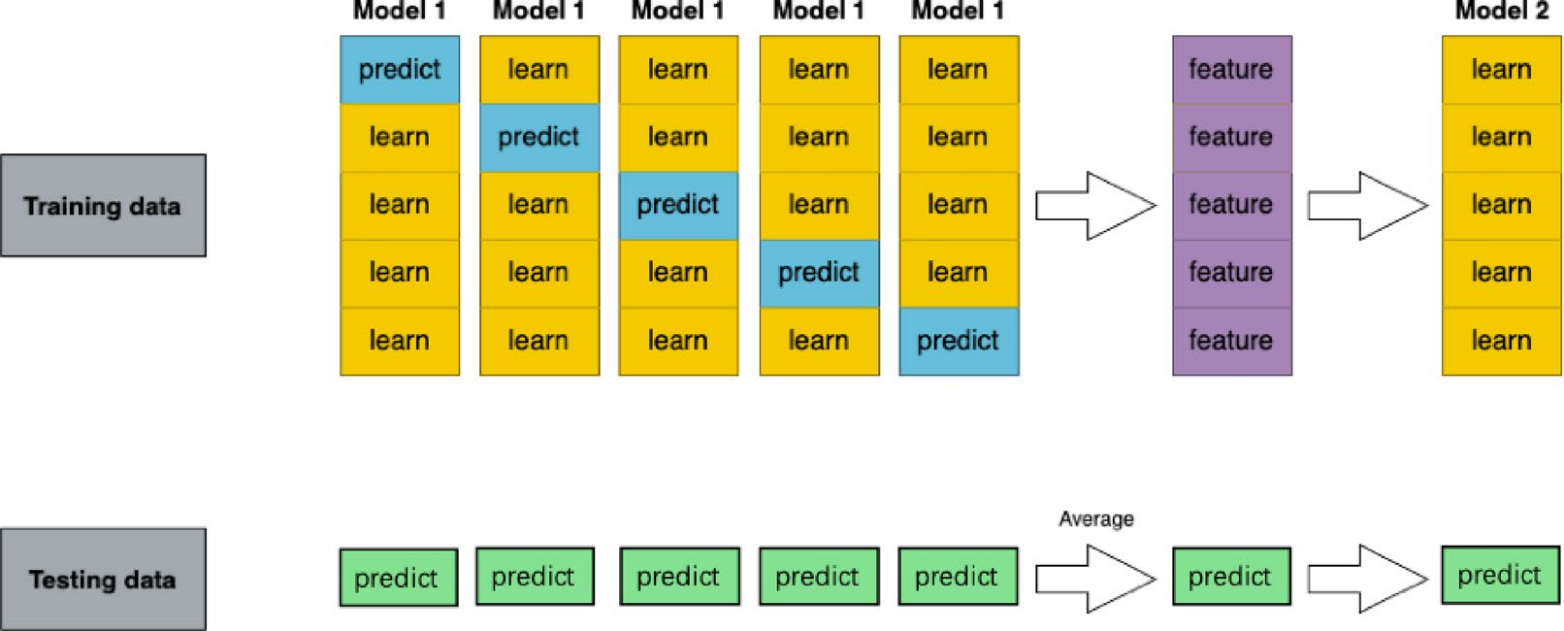
Training Process of the Stacking Ensemble Learning Model.

The Stacking ensemble framework can be formally defined as follows: Given a k-fold cross-validation setup, let (*x*,* y*)*k* represent the k data folds, *x* = (*x₁*, *x₂*, …, *xr*) corresponds to the r recorded feature values, and *y* = (*x*_*r*+1_, *x*_*r*+2_, …, *x*_*r+p*_) represents the p target values to be predicted. For a given set of N potential learning algorithms, denoted as *modeli*, *i* = *1*, …, *N*, Let *A*_*ij*_ represent the model created by algorithm *modeli* trained on x to predict *x*_*p+j*_. The generalizer function, denoted as *G*_j_, is responsible for merging predictions from the base models to produce the final forecast. *G*_j_ can be either a meta-model trained via a learning algorithm or a general function such as a weighted average. Thus, the estimated prediction value $\hat{x}$ *(p + j)* can be formally expressed by Eq. 4. The training process is shown in Fig. 3. The effectiveness of the Stacking ensemble learning method relies on two key factors: (1) the performance of the base classifiers—the better the performance of the base models, the higher the overall ensemble performance; and (2) the diversity among the base classifiers. Introducing heterogeneous base models allows each to contribute uniquely, improving the ensemble model’s generalization capability.

4 $$\:\hat{x}\left( {p + j} \right) = G_{j} (A_{{ij}} , \ldots \:,A_{{Nj}} )$$

In this study, the stacked ensemble method was constructed by selecting six classifiers—XGBoost, KNN, AdaBoost, Naive Bayes, Logistic Regression, and Decision Tree—from multiple candidate models based on their highest cross-validation accuracy, which served as the base layer of the ensemble model. At the second layer, a MLP was used as the meta-learner (super learner). The MLP consisted of two hidden layers, each comprising 50 neurons, and was responsible for aggregating the prediction outputs from the base classifiers to produce the final prediction.

**Fig. 4:**
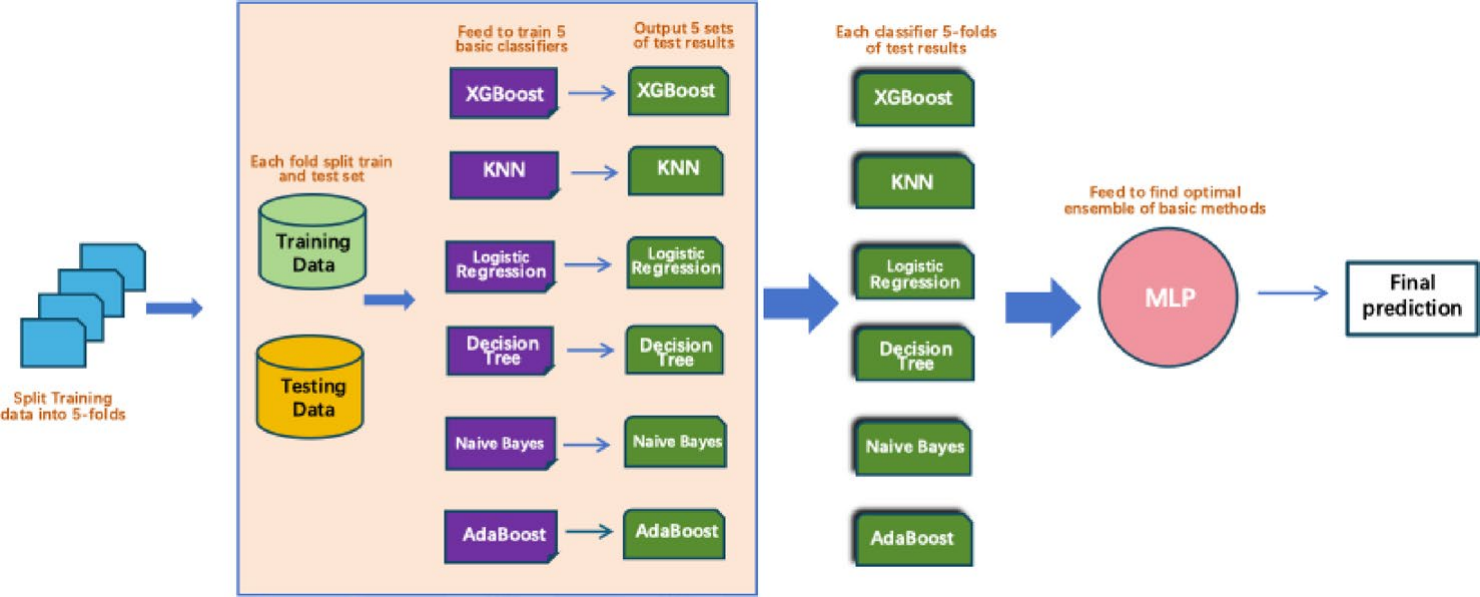
Complete the Training Process of the Stacking Model with 5-Fold Cross-Validation.

The complete training process is illustrated in Fig. 4 and follows a 5-fold cross-validation scheme. Specifically, the training data were randomly divided into five equally sized folds. In each iteration, four folds were used for training and the remaining fold served as the validation set. This process was repeated five times, ensuring that each fold functioned as the validation set once. During each fold, all six base classifiers were trained on the training subset and used to generate predictions on the corresponding validation fold. This resulted in five prediction sets per classifier. These out-of-fold predictions were then concatenated to form a new feature matrix, which served as the input to the meta-learner for training.

Simultaneously, predictions were made on the test set in each fold using the base classifiers, and the average of these predictions was used to construct the test set input for the meta-learner. Finally, the MLP meta-classifier used these new features to produce the final prediction results.

#### Interpretability analysis

The model’s predictions are analyzed using SHAP (Shapley Additive Explanations) values, which measure the contribution of each feature to the output for a specific instance. SHAP is grounded in cooperative game theory, where the model prediction is treated as a total reward, and the feature values are considered players contributing to this reward^30^.

Consider a dataset that contains *p* features, represented by the set *F = {1*,* 2*,* 3*,* …*,* p}*. A subset of features *C ≤ F* is referred to as a coalition, representing a collective contribution to the prediction. The empty set, denoted as $\:\tau\:$, represents a scenario with no contributing features. A characteristic function *v* is defined to assign a real value to each subset *C*, such that *v(C)* reflects the predictive value of the coalition. To determine the individual contribution of feature *i*, SHAP evaluates all possible permutations of the feature set and computes the average marginal contribution of feature *i* across all coalitions that exclude *i*. The SHAP value $\:\tau\:$_*i*_ for feature *i* is formally defined in Eq. 5.

5 $$\:{\tau\:}_{i}=\frac{1}{\left|F\right|!}\:\sum\:_{p}\left(\nu\:\left(C\cup\:\left\{i\right\}\right)-\nu\:\left(C\right)\right)\:$$

We utilize SHAP (Shapley Additive Explanations) analysis to enhance the interpretability of our NBA game prediction model. Specifically, we apply Kernel SHAP to perform both Global Mean Analysis and Local Interpretability, enabling us to quantify the influence of individual features on the model’s predictions across different scenarios.

##### Global interpretability

Global interpretability assesses how individual features influence the model’s predictions on average. The horizontal axis represents the average change in model output when a specific feature is omitted or “masked.” In this context, “masked” signifies that the feature is removed from the input, thereby eliminating its influence on the prediction. Let *N* denote the total number of samples, $\:{\varphi\:}_{i}^{\left(j\right)}$ represent the SHAP value of the *i*-th feature for the *j*-th sample, and $\:{\varPhi\:}_{i}^{global}$denote the global average SHAP importance of the *i*-th feature. The Global SHAP value can be represented using Eq. 6 as follows:

6 $$\:{{\Phi\:}}_{i}^{global}=\:\frac{1}{N}\sum\:_{j=1}^{N}\left|{\varphi\:}_{i}^{\left(j\right)}\right|\:\:\:\:$$

##### Local interpretability

Local interpretability explains individual predictions, analyzing key factors driving specific NBA game outcomes. Where f(x): the model’s prediction output for sample *x*; ∅_*0*_ the model’s baseline output (typically the expected output over background data); ∅_*i*_ the SHAP value of feature *i* for the given sample, representing the individual contribution of feature *i* to the prediction; *p* the total number of features. The Local SHAP Approximation Model can be represented using Eq. 7 as follows:

7 $$\:f\left(x\right)\approx\:\varphi\:+\sum\:_{i=1}^{p}{\varnothing\:}_{i}$$

#### Performance matrix

This study applies multiple machine learning models, with their effectiveness evaluated through performance metrics: Accuracy, Precision, Recall, and F1-score, each formally described in Eqs. 8, 9, 10, and 11^31^. In this context, TP, FP, TN, and FN represent True Positives, False Positives, True Negatives, and False Negatives, respectively. In addition to these conventional metrics, the Receiver Operating Characteristic - Area Under the Curve (ROC-AUC) is used to evaluate classification quality. The ROC curve depicts the relationship between the True Positive Rate (TPR), which is the same as Recall, and the False Positive Rate (FPR) as defined in Eqs. 9 and 12. This curve offers a comprehensive visualization of a model’s diagnostic ability across all possible classification thresholds, allowing for an assessment of its overall discriminative power.

8 $$\:\text{A}\text{c}\text{c}\text{u}\text{r}\text{a}\text{c}\text{y}=\:\frac{\left(TP+TN\right)}{\left(TP+TN+FN+FP\right)}\:\:\:\:$$

9 $$\:\text{P}\text{r}\text{e}\text{c}\text{i}\text{s}\text{i}\text{o}=\:\frac{\left(TP\right)}{\left(TP+FP\right)}\:\:$$

10 $$\:Reca=\:\frac{\left(TP\right)}{\left(TP+FN\right)}\:$$

11 $$\:\:F1\:scor=\frac{\left(2*Precision*Recall\right)}{\left(Precision+Recall\right)}\:$$

12 $$\:FPR=\:\frac{\left(FN\right)}{(TP+FN)}\:$$

## Results

This section presents an in-depth evaluation of the model’s performance. Various performance indicators—accuracy, precision, recall, F1-score, and the area under the ROC curve (AUC)—are utilized to assess the model’s predictive capability. These metrics are calculated using the test dataset to ensure an unbiased assessment of the model’s generalization performance. To better understand the model’s behavior, SHAP values are utilized to provide insights into the contributions of individual features to the prediction process, helping to identify key factors that influence NBA game outcomes. A holistic view of the model’s reliability and decision logic is achieved by combining quantitative performance analysis with interpretability methods. This rigorous evaluation underscores the model’s potential in sports analytics and demonstrates its relevance in practical, competitive basketball scenarios.

### Model training and experimental results

The experimental setup was implemented on a machine powered by an 8th-generation Intel Core i7 CPU running at 3.1 GHz with 16 GB of RAM. To improve the model’s performance, we conducted hyperparameter optimization using the GridSearchCV method.

Table 6 summarizes the results of baseline models and the proposed stacking model (with an MLP as the meta-learner). Among all baseline models, AdaBoost (81.10%), XGBoost (81.03%), and Logistic Regression (80.49%) achieved relatively high prediction accuracy. KNN (80.15%) and Naive Bayes (76.56%) performed moderately well, while the Decision Tree (74.19%) yielded the lowest accuracy, reflecting limited stability and generalizability. In contrast, the Stacking model (MLP) achieved the best overall performance, with an accuracy of 83.27%, an F1-score of 0.8350, and an AUC of 0.9213, outperforming all individual models.

**Table 6:** Performance of machine learning algorithm.

Classic	Accuracy (%)	Precision (%)	Recall (%)	F1 Score (%)	ROC (%)
XGBoost	81.03	82.36	80.18	81.26	90.82
KNN	80.15	81.18	79.79	80.48	87.92
Decision Tree	74.19	75.07	74.37	74.72	79.87
AdaBoost	81.10	81.53	81.64	81.58	89.97
Logistic Regression	80.49	81.90	79.52	80.70	90.28
Naïve Bayes	76.56	79.15	73.71	76.33	85.67
Sacking (MLP)	83.27	84.46	82.56	83.50	92.13

**Fig. 5:**
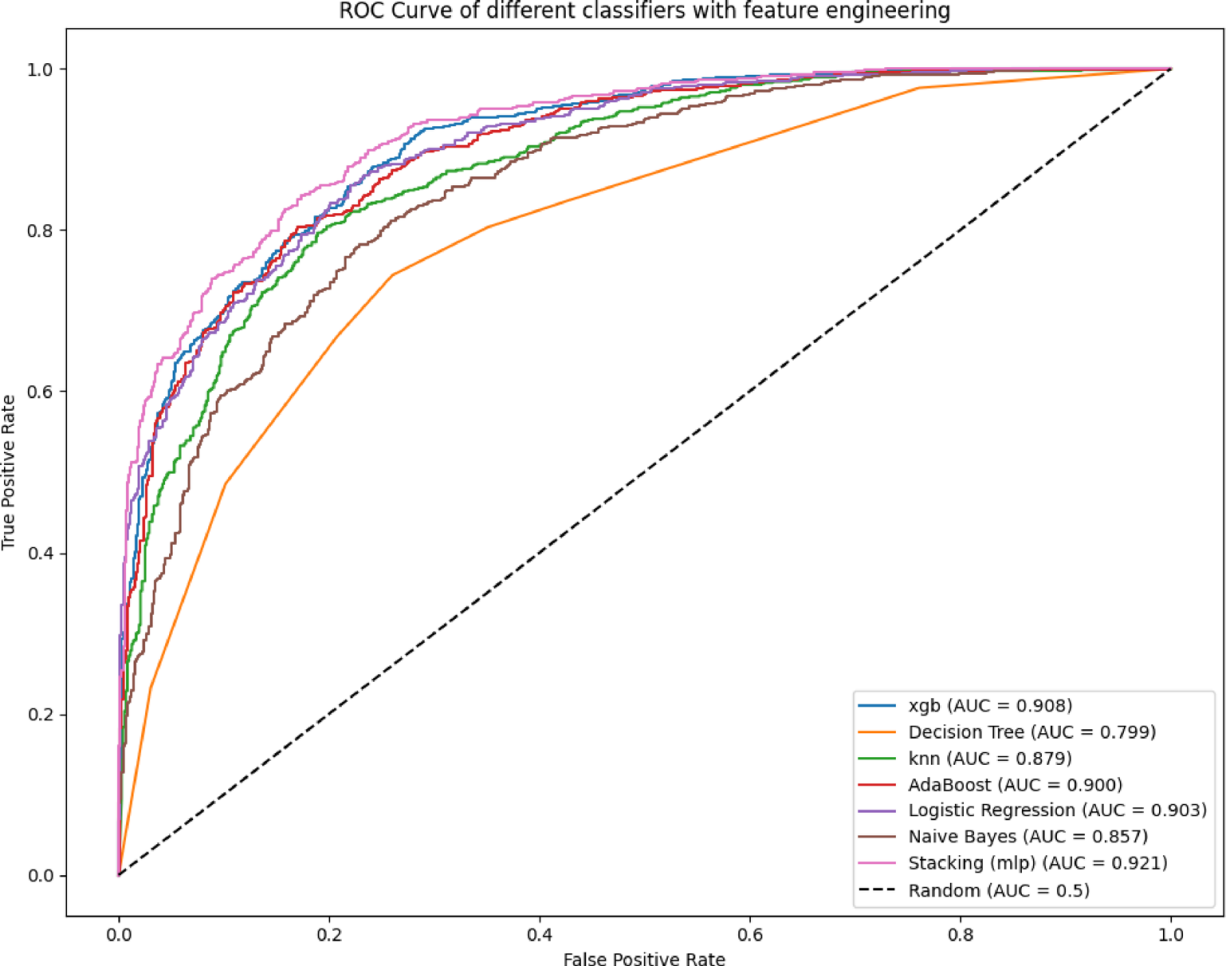
ROC Curves and AUC Scores of All Models on the Test Set.

Figure 5 illustrates the ROC curves of all models evaluated on the test set, with the AUC values annotated in the legend. A curve closer to the top-left corner indicates stronger classification ability. The Stacking model achieves the highest AUC (0.921), indicating superior discriminative performance. XGBoost (0.908), Logistic Regression (0.903), and AdaBoost (0.900) also demonstrate strong performance. In contrast, KNN (0.879) and Naive Bayes (0.857) show moderate effectiveness, while the Decision Tree model (0.799) lags significantly. A random classifier baseline (AUC = 0.5) is included for reference. These results demonstrate the effectiveness of ensemble strategies, particularly Stacking, in improving overall model performance by leveraging the strengths of multiple base learners.

**Fig. 6:**
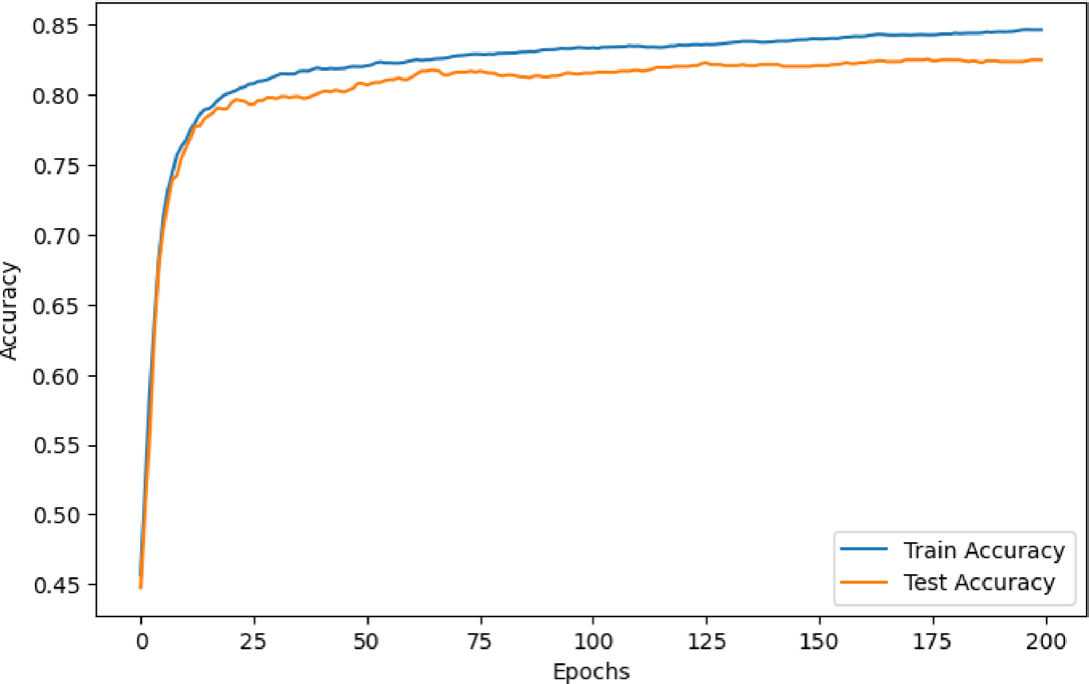
Learning Curve of the Stacking Model: Training vs. Testing Accuracy.

**Fig. 7:**
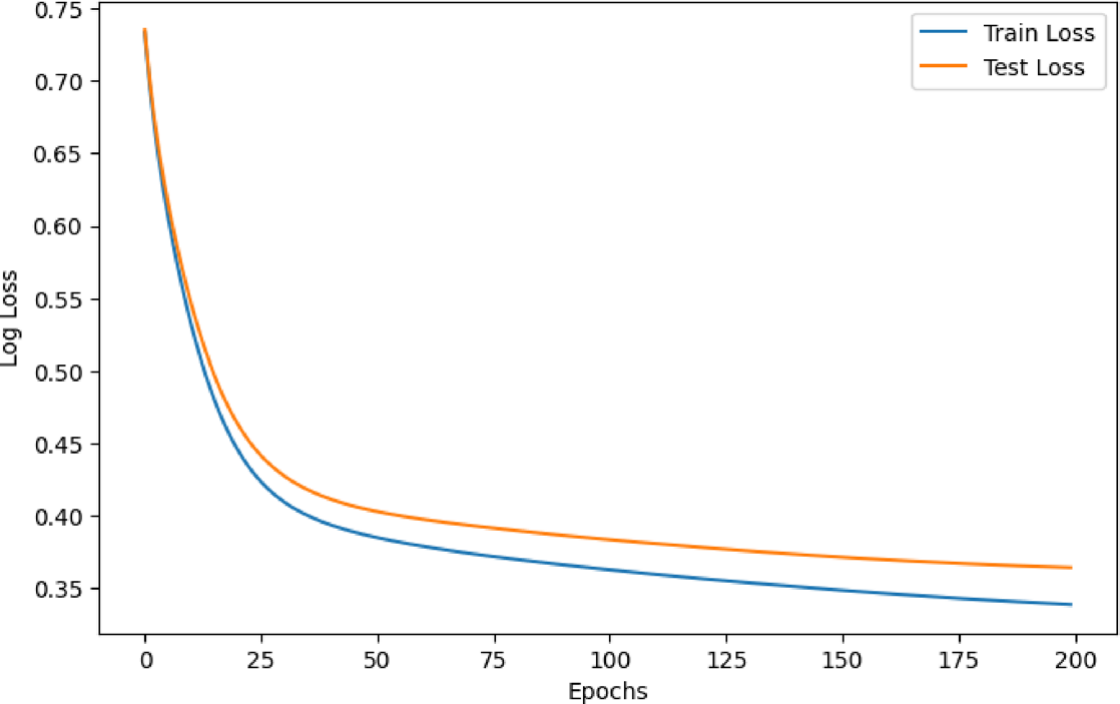
Learning Curve of the Stacking Model: Training vs. Testing Loss.

To further evaluate the training dynamics and assess the presence of potential overfitting or underfitting, learning curves for the stacking model were plotted, as shown in Figs. 6 and 7. Figure 6 illustrates the training and testing accuracy over 200 epochs. Both curves show a rapid increase in the early stages and gradually converge, with only a slight gap between training and testing accuracy, suggesting good generalization and minimal overfitting. Figure 7 presents the corresponding log loss curves for both the training and testing sets. The loss decreases steadily and smoothly across epochs for both sets, with no divergence. The similar downward trend of both curves confirms that the model maintains a consistent learning process without significant variance issues. These results further support the robustness and stability of the proposed ensemble framework.

After obtaining the prediction accuracies of the various models, statistical hypothesis testing was conducted to determine whether the observed performance differences were statistically significant. Specifically, a paired t-test was performed to compare the classification accuracies of each model under identical cross-validation folds. As shown in Table 7, the results indicate that the stacking model significantly outperforms all individual baseline classifiers in terms of classification accuracy (*p* < 0.05). Although ensemble methods such as XGBoost and AdaBoost achieved relatively high accuracy, the differences in performance compared to the stacking model were still statistically significant (*p* = 0.0379 and *p* = 0.0037, respectively). This underscores the added value of the stacking approach, which effectively leverages the complementary strengths of multiple learners to deliver superior predictive performance. Traditional classifiers such as KNN and Decision Tree exhibited substantial performance gaps (*p* < 0.0001), further validating the effectiveness of the stacking ensemble in improving both generalization ability and classification accuracy for complex tasks such as game outcome prediction.

**Table 7:** Paired t-Test results comparing model accuracies with the stacking Ensemble.

Model	Mean Accuracy	t-Statistic	*p*-value
KNN	0.788	−21.942	< 0.001
Decision Tree	0.752	−18.321	< 0.001
Naive Bayes	0.774	−13.407	< 0.001
Logistic Regression	0.819	−10.470	< 0.001
AdaBoost	0.822	−6.097	0.0037
XGBoost	0.828	−3.053	0.0379

### SHAP-based interpretability analysis

To improve the interpretability of the black-box model, SHAP values are calculated for the stacking model, which is MLP-based. A representative sample of 300 test instances is selected to analyze individual features’ contributions and directional impacts. SHAP provides a powerful and intuitive framework for quantifying how each feature influences the model’s prediction, allowing for a better understanding of the most influential features in-game outcome predictions.

#### Global feature importance

**Fig. 8:**
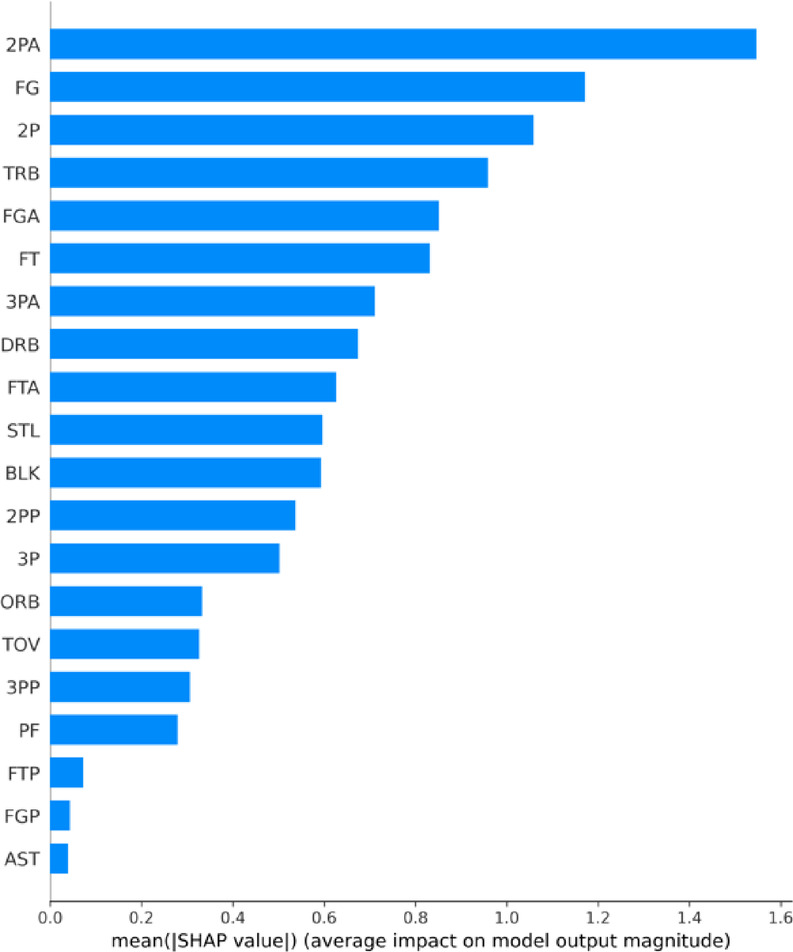
Mean SHAP Values Indicating Feature Importance in the Stacking Model.

Figure 8 visualizes the mean SHAP value of each selected feature. The x-axis indicates the average impact magnitude, while the y-axis lists features ranked by their contribution to the model’s output. The most important features are 2PA (two-point attempts), FG (field goals made), 2P (two-point makes), and TRB (total rebounds), whereas AST (assists) appears to have minimal influence. These results indicate that shooting frequency, accuracy, and rebounding influence game outcomes.

**Fig. 9:**
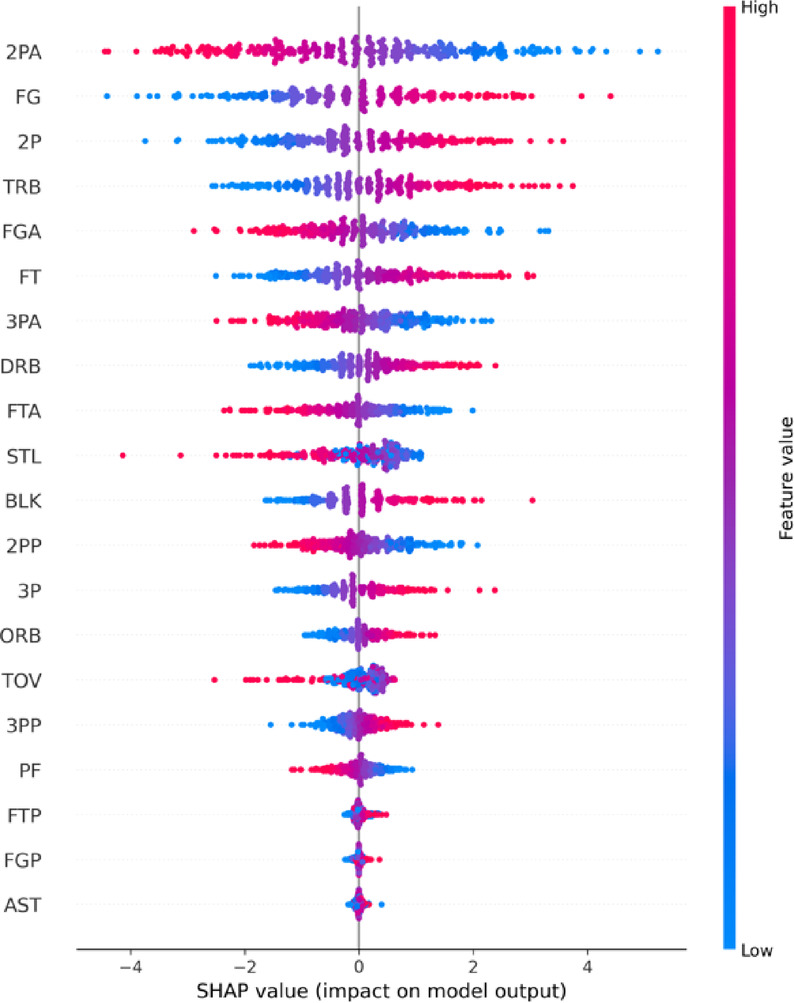
SHAP Summary Plot Showing Feature Value Impact on Prediction Direction.

As a complement, Fig. 9(SHAP Summary Plot) further illustrates how high and low feature values affect prediction direction. The SHAP values are plotted along the x-axis, and feature values are color-coded from red (high) to blue (low). The results show that higher values of FG and TRB positively influence the win prediction. In contrast, higher FGA and 2PA negatively affect the outcome, suggesting that high-volume but low efficiency shooting teams are likelier to lose. In particular, the negative impact of 2PA warrants closer attention. Although two-point shots are generally taken closer to the basket and theoretically pose a higher scoring threat, they are also more susceptible to defensive interference. In practice, defenders often prioritize rim protection, which reduces the effectiveness of frequent inside shot attempts. As a result, a high number of two-point attempts may reflect a “high volume, low conversion” pattern of inefficient offense. Furthermore, previous studies have observed that a surge in two-point attempts often occurs when teams are trailing, representing a reactive rather than strategic offensive behavior—one that is frequently associated with game losses^32,33^. Consequently, the model likely learns that when high 2PA is not accompanied by sufficient shooting efficiency (e.g., high field goal percentage), it acts as a negative signal, thereby contributing adversely to the predicted outcome.

#### Feature interaction analysis

**Fig. 10:**
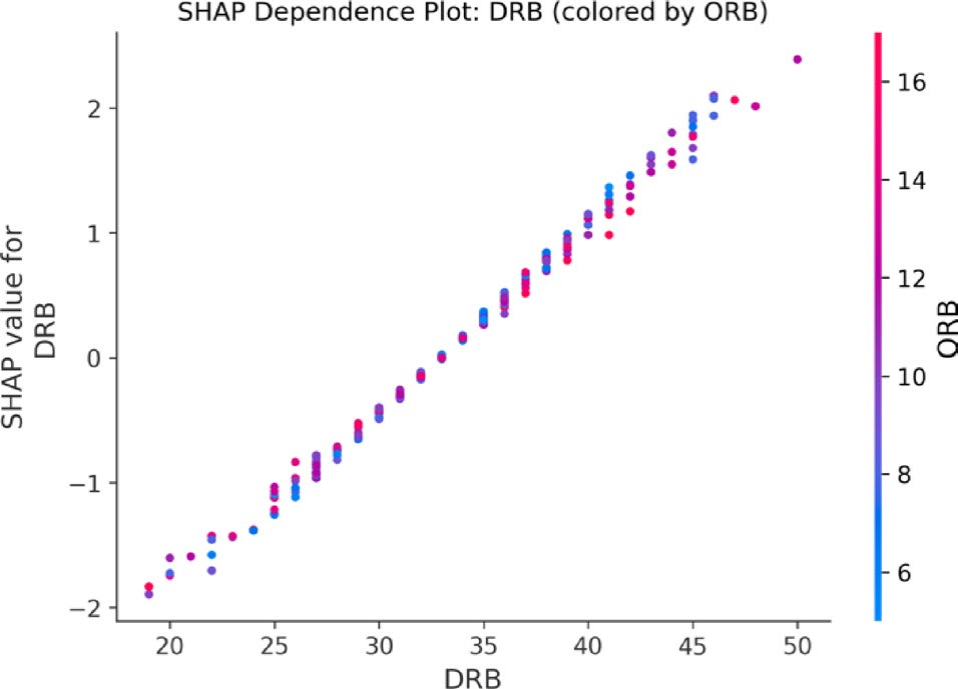
Interaction Effect Between Defensive and Offensive Rebounds Based on SHAP Values.

Figure 10 visualizes the interaction between DREB and OREB. The left y-axis corresponds to SHAP values, while the right y-axis indicates scaled OREB values. The data points are color-coded based on OREB values (red for high, blue for low). When DREB values are high, the SHAP values increase further under high OREB conditions. This suggests a synergistic effect between offensive and defensive rebounding, reinforcing that these features collectively drive better game outcomes.

#### Individual SHAP explanation

To further understand the prediction mechanism at the individual level, we apply SHAP to interpret a single test sample predicted as a loss (model output f(x) = −1.058).

**Fig. 11:**
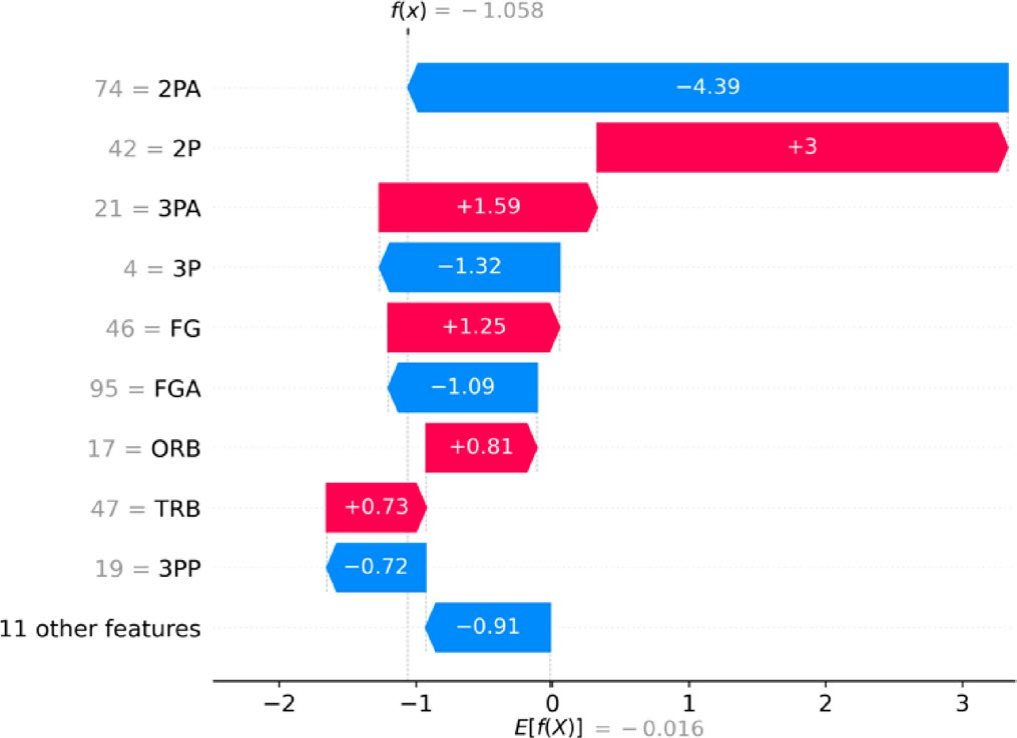
SHAP Waterfall Plot Showing Feature Contributions to a Single Loss Prediction.

Figure 11 (Waterfall Plot) quantifies each feature’s contribution to the prediction outcome: 2PA = 74 had the most negative impact (SHAP = −4.39), suggesting many inefficient shot attempts. 2P = 42 and FG = 46 had substantial positive contributions (+ 3.00 and + 1.25, respectively), reflecting good scoring performance. TRB (47) and ORB (17) also positively impacted the prediction score. Conversely, FGA = 95 and 3P = 4 contributed negatively, indicating lower shooting quality.

**Fig. 12:**
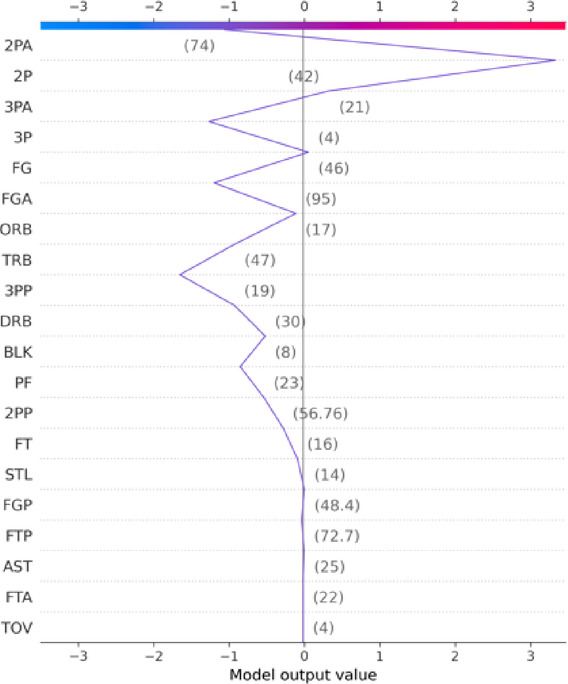
SHAP Decision Plot of Cumulative Feature Contributions.

Figure 12 (Decision Plot) visualizes the cumulative path from the model’s baseline output to the final prediction. Features like 2PA, FGA, and 3P caused a sharp decrease in prediction value, while 2P, FG, and TRB contributed to a slower, stabilizing rise. This plot offers insight into the model’s logical decision-making progression.

**Fig. 13:**
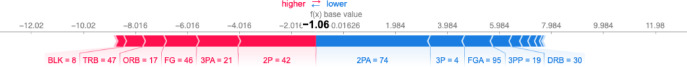
SHAP Force Plot of Feature Impact on Model Prediction.

Figure 13 (Force Plot) provides a comprehensive visualization of how all features jointly affect the final prediction. Red arrows indicate features pushing the prediction higher, while blue arrows indicate negative influences. The length of each arrow represents the strength of the contribution. This figure underscores the balance of competing factors influencing the model’s decision and enhances interpretability for end users.

## Discussions

This study proposes a hybrid modeling framework integrating ensemble learning with model interpretability mechanisms to predict team outcomes in NBA games. The overall predictive performance is significantly improved by introducing a stacking ensemble model with an MLP meta-learner and combining multiple base classifiers (such as XGBoost, AdaBoost, Logistic Regression, etc.). Experimental results show that the proposed method surpasses all individual models in accuracy, F1-score, and AUC, achieving the highest test accuracy of 83.60%. This underscores its robust generalization ability and real-world applicability.

To further enhance interpretability, we adopt SHAP to analyze the model’s decision logic at both global and individual levels. Globally, feature importance analysis reveals that two-point attempts (2PA), field goals made (FG), total rebounds (TRB), and field goal attempts (FGA) are the most influential factors affecting game outcomes. At the individual level, the model’s inference process is visualized through Waterfall and Force plots, illustrating each feature’s positive or negative contribution to the final prediction.

Beyond individual feature impact, this study also explores feature interactions. The SHAP dependence plots indicate a significant synergistic effect between defensive rebounds (DRB) and offensive rebounds (ORB), confirming that the model captures complex inter-feature relationships. These interpretability results improve model transparency and offer actionable insights for lineup optimization and tactical design.

Despite these promising outcomes, several limitations remain. First, although SHAP can reveal feature interactions, it cannot quantitatively model interaction strength. Future work could incorporate graph neural networks (GNNs) or self-attention mechanisms better to capture high-order feature interactions in a structured manner. Second, the current model is trained on structured game statistics without including unstructured or dynamic data such as player trajectories, spatial positioning, or tactical shifts. These modalities further enhance the model’s understanding of in-game dynamics and real-time adaptability. Finally, as the dataset used is based on NBA games, the model’s generalizability across different leagues (e.g., CBA, NCAA) or other team sports (e.g., soccer, volleyball) warrants further validation.

In conclusion, this study confirms AI models’ substantial potential in combining ensemble learning with interpretability techniques in competitive sports analytics. The proposed model exhibits strong predictive power and provides coaches and decision-makers with interpretable, visual, and tactical insights. As sports data becomes more complex and analysis tools continue to improve, artificial intelligence is set to play a crucial role in match simulation, tactical optimization, substitution strategies, and lineup planning. This shift will ultimately advance competitive sports toward a more data-driven and intelligent approach.

## Conclusion and future work

This research introduces an AI-driven strategy to improve the predictive accuracy and interpretability of NBA game outcome models. The results underscore machine learning methodologies’ promising role in sports science, particularly for forecasting results in elite-level basketball competitions. A key contribution of this study is implementing a Stacked Ensemble Learning approach. This method leverages the unique strengths of various base classifiers to enhance predictive performance. The proposed ensemble framework attained an accuracy of 83.96%. Surpassing individual model benchmarks reinforces the value of ensemble techniques in sports analytics.

Overall, the core contribution of this study lies in the design and validation of an efficient and interpretable hybrid modeling framework. It empirically demonstrates the performance advantages of ensemble learning in the context of sports prediction. It effectively utilizes interpretability analysis tools to enhance the understanding of model outputs while supporting their practical application in real-world scenarios. Future research could integrate deep learning techniques to optimize model architecture and explore more complex feature interactions. Additionally, incorporating additional data sources (e.g., player fitness levels, tactical strategies, and in-game dynamics) or leveraging advanced techniques such as reinforcement learning may improve the transparency and reliability of NBA game prediction models^34–41^.

Moreover, the current model—while trained on NBA datasets—has the potential for transfer to other basketball leagues (e.g., NCAA, CBA, Euro League) or even different team sports such as soccer, baseball, or volleyball. The modeling pipeline is highly modular and can be retrained on sport-specific features. With the increasing availability of real-time match data, future work may also explore the application of this framework to live prediction and in-game strategy adjustment. The integration of predictive analytics into tactical systems could enable intelligent decision support for coaches, advancing the role of AI in competitive sports environments.

## Supplementary Information

Below is the link to the electronic supplementary material.

Supplementary Material 1

## Data Availability

The dataset used in this study is publicly available from the official NBA website: https://www.nba.com.
